# Fatty acid-binding protein 4 predicts gestational hypertension and preeclampsia in women with gestational diabetes mellitus

**DOI:** 10.1371/journal.pone.0192347

**Published:** 2018-02-02

**Authors:** Boya Li, Huixia Yang, Wanyi Zhang, Yundi Shi, Shengtang Qin, Yumei Wei, Yingdong He, Wenshuang Yang, Shiju Jiang, Hongyan Jin

**Affiliations:** 1 Peking University First Hospital, Beijing, China; 2 Department of Pharmacology, School of Basic Medical, Peking University Health Science Center, Beijing, China; Shanghai Diabetes Institute, CHINA

## Abstract

**Objective:**

Fatty acid-binding protein 4 (FABP4) has been proposed to be a potential predictive factor of gestational hypertension or preeclampsia (GH/PE) because of its integrating metabolic and inflammatory responses. Women with gestational diabetes mellitus (GDM) are more likely to develop both GH/PE, than the normal population. The aim of our study was to examine the relationship between plasma FABP4 in the second trimester of pregnancy and the risk of GH/PE in women with GDM.

**Methods:**

This was a nested case-control study conducted within a large on-going prospective cohort study conducted at Peking University First Hospital. A total of 1344 women, who were diagnosed with GDM, according to a 75 g oral glucose tolerance test, participated in the GDM One-Day Clinic at Peking University First Hospital from February 24, 2016 to February 9, 2017. Of the 748 GDM women who agreed to the blood sample collection, 637 were followed until their delivery. The cases included GDM patients who developed gestational hypertension or preeclampsia (GDM-GH/PE group, n = 41). Another 41 matched GDM women without major complications were selected as the control group (GDM group).

**Results:**

The incidence of GH/PE was 6.44% and 3.30% for preeclampsia. The level of the second trimester plasma FABP4 in the GDM-GH/PE group was significantly higher than the GDM group (17.53±11.35 vs. 12.79±6.04 ng/ml, P = 0.020). The AUC ROC for the second trimester plasma FABP4 predicted GH/PE in the GDM patients alone was 0.647 (95%CI 0.529–0.766). Multivariate analysis showed that the elevated second trimester FABP4 level was independently associated with GH/PE in the GDM patients (OR 1.136 [95% CI 1.003–1.286], P = 0.045).

**Conclusions:**

Increased second trimester plasma FABP4 independently predicted GH/PE in GDM patients.

## Introduction

Gestational hypertension and preeclampsia (GH/PE) are characterized by insulin resistance and inflammation. Women with these diseases have an increased risk of developing metabolic syndrome and cardiovascular disease in the future.

Fatty acid binding protein-4 (FABP4), also known as adipocyte fatty acid binding protein (AFABP), has recently been suggested as a third adipokine, in addition to leptin and adiponectin [1]. It was first found produced in adipocytes and is released into circulation [1]. Recent studies showed that trophoblasts, macrophages and endothelial cells also express FABP4 [2–4]. Thus, it may be involved in glucose and lipid metabolism, inflammation, insulin resistance, and other aspects.

In the non-pregnant state, FABP4 is associated with risk factors of preeclampsia, such as obesity, hypertension, and diabetes mellitus [5]. Previous studies revealed that it is not only an independent risk factor for metabolic syndrome and atherosclerosis [6, 7] but also a direct cause of these diseases [8–10]. A recent study reported elevated serum FABP4 levels in women with preeclampsia [11]. It was then proposed to be a potential predictive factor of preeclampsia or pregnancy induced hypertension in pregnant women with different characteristics (healthy, overweight and women with type 1 diabetes) [11–13].

Gestational diabetes mellitus (GDM) is associated with the increase of insulin resistance as pregnancy progresses. Women with GDM are more likely to develop both GH/PE than the normal population. In this article, we examined the level of plasma FABP4 in the second trimester of pregnancy in GDM women to test the hypothesis that FABP4 is a potential predictive factor of subsequent GH/PE. We also investigated the influence of the state of glucolipid metabolism and insulin resistance on FABP4 and the development of GH/PE.

## Methods

### Participants

This was a nested case-control study conducted within a large on-going prospective cohort study conducted at Peking University First Hospital of Beijing designed to investigate the effects of glucose metabolism, lipid metabolism and insulin resistance on pregnancy outcome in GDM women. A total of 1344 women, who were diagnosed with GDM, according to a 75 g oral glucose tolerance test, participated in the GDM One-Day Clinic at Peking University First Hospital from February 24, 2016 to February 9, 2017. Of the 748 GDM women who agreed to the blood sample collection, 637 were followed until their delivery. The exclusion criteria included the following: gestational age > 32 weeks of pregnancy, pre-pregnancy diabetes and chronic kidney disease. The cases included GDM patients that developed gestational hypertension or preeclampsia (GDM-GH/PE group, n = 41). Another 41 matched GDM women without major complications were selected as the control group (GDM group) according to their age, gestational weeks, and pre-pregnancy BMI. Specifically, hypothyroidism, Hashimoto's thyroiditis, and allergic asthma were acceptable complications in addition to a diagnosis of GDM.

### Diagnostic criteria

GDM was diagnosed with the “One-step” approach (first proposed by the IADPSG [14] and recognized by the Chinese association of perinatal medicine [15]). The 75-g OGTT was performed the morning after an overnight fast of at least 8 h and plasma glucose was measured fasting and at 1 and 2 h after 24 weeks of gestation in women without overt diabetes. GDM diagnosis is made when any of the following plasma glucose values are exceeded: Fasting: 5.1 mmol/L; 1 h: 10.0 mmol/L; 2 h: 8.5 mmol/L.

Preeclampsia occurs as new hypertension beginning after 20 weeks with significant proteinuria. In the absence of proteinuria, preeclampsia is diagnosed accompanied by any of the following organs or system involvement: thrombocytopenia, impaired liver function, the new development of renal insufficiency, pulmonary edema, or new-onset cerebral or visual disturbances [16, 17]. Gestational hypertension occurs as new-onset hypertension (SBP ≥140 mmHg or DBP≥ 90 mmHg) starting after 20 weeks without proteinuria [16, 17]. Chronic hypertension is hypertension diagnosed before 20 weeks of gestation [16, 17]. Chronic hypertension superimposed preeclampsia (CH superimposed PE) is chronic hypertension in association with preeclampsia [16, 17].

Chronic kidney disease is a type of kidney disease in which there is gradual loss of kidney function over a period of months or years, manifested as hematuria, proteinuria with or without decreased glomerular filtration rate. In our study, chronic kidney disease refers to IgA nephropathy, chronic glomerulonephritis, occult nephritis, pyelonephritis, purpura nephritis, lupus nephritis, etc.

### Clinical and biochemical assessments

Clinical data, including maternal age, height, weight at the first study visit, pre-pregnancy weight, OGTT results, and second trimester systolic and diastolic blood pressure, were collected prospectively from the patient’s medical record. Mean arterial pressure (MAP) was calculated as ([2DBP] +SBP)/3. Pre-pregnancy body mass index (BMI) was calculated as pre-pregnancy weight (kilograms) divided by squared height (meters). Second trimester BMI was calculated as weight at the first study visit (kilograms) divided by squared height (meters).

Fasting blood glucose, triglyceride (TG), total cholesterol (TCHO), high-density lipoprotein (HDL), low-density lipoprotein (LDL), insulin, and glycosylated hemoglobin A1c (HbA1c) levels were measured after 8 h of fasting at 24–32 wks when they first went to the GDM One-Day Clinic. Homeostasis model assessment-insulin resistance (HOMA-IR) was used as an index to evaluate the level of individual insulin resistance. HOMA-IR was calculated as fasting blood glucose (mmol/L) × fasting insulin (mIU/L) /22.5. With the increase of insulin resistance levels, HOMA-IR will increase accordingly.

Heparin anticoagulated plasma was centrifuged and stored in a -80°C freezer until analysis. Plasma FABP4 concentrations were analyzed blinded to their preeclampsia status with BioVendor Human AFABP ELISA (Cat. No: RD191036200R, Biovendor, Modrice, Czech Republic). The manufacturer reports a normal range of 19.58 ± 16.32 ng/mL (mean ± 2 SD) for 35- to 52-year-old women. The limit of detection of the FABP4 ELISA assay is 0.05 ng/ml. The antibodies used in this ELISA are specific for human AFABP according to the manufacturer. The detail of the laboratory protocols can be find from http://dx.doi.org/10.17504/protocols.io.md9c296.

The authors had access to information that could identify individual participants during or after data collection.

### Ethics and data availability statement

The study was reviewed and approved by the Institutional Review Board of the First Hospital, Peking University (Reference number: 2013[572]). All participants provided written informed consent, and the Ethics Committee approved the consent procedure. All relevant data are within the paper.

### Statistical analysis

Normally distributed data are given as the means ± SDs and non-normally distributed data as medians with ranges. Kolmogorov-Smirnov test was used in testing for normal distribution. Independent sample t test comparison was used for data to determine the normal distribution and homogeneity of variance. Nonparametric test (Mann-Whitney test) was used for data with non-normal distribution. Correlations were performed by using the spearman’s rank correlation method. Logistic regression analysis (backward condition method) was used to investigate the relationship between FABP4 and GH/PE in GDM women before and after adjusting for the following risk factors: age, pre-pregnancy BMI, second trimester BMI, gestational age, TG, TCHO, HDL, LDL, HbA1c, HOMA-IR, and MAP. Collinear diagnosis was performed using Variance inflation factor (VIF) and tolerance before the multivariate analysis. Collinearity was considered strong when VIF > 10 or tolerance <1, which implicated the need for variable merge. The area under the receiver operating characteristic curve (AUROC) was also calculated to assess the predictive value of FABP4 alone and the multi-factor model for GH/PE. P values of <0.05 were considered statistically significant in all analyses. Statistical analyses were performed using SPSS 19.0 software.

## Results

### Baseline and metabolic characteristics

Twenty-one patients developed gestational hypertension and twenty developed preeclampsia (3 were early-onset and 17 were late-onset), from the 637 GDM patients who were followed until their delivery. The incidence of GH/PE was 6.44% (41/637) and 3.30% for preeclampsia (20/637). One patient with GH and four with preeclampsia had complications with fetal growth restriction. Four patient in the GH/PE group were chronic hypertension superimposed with preeclampsia, and the three of them taking antihypertensive drugs since the first trimester.

The baseline and metabolic characteristics of the GDM-GH/PE group and the GDM group are summarized in Table 1. There were no significant differences in age, gravidity, parity, pre-pregnancy BMI, second trimester BMI, gestational age, OGTT, and HbA1c levels between the two groups. The SBP and DBP in the GDM-GH/PE group were 131 ± 10 mmHg and 78 ± 9 mmHg, respectively, and the MAP was 95.5 ± 8.0 mmHg, which was significantly higher than that of the GDM group (SBP and DBP were 118 ± 13 mmHg and 68 ± 9 mmHg; MAP was 85.1 ± 9.6 mmHg; P <0.001). The HOMA-IR in the GDM-GH/PE group was significantly higher than that of the GDM group (3.37 ± 1.71 vs. 2.54 ± 1.54, P = 0.024). In the comparison of their lipid metabolism, TG was significantly higher in the GDM-GH/PE group than the control group (2.60 [quartile range 2.30–2.98] vs. 3.13 [2.43–3.85] mmol/l, P = 0.017). However, there was no significant difference in TCHO, HDL and LDL levels between the two groups.

**Table 1:** Baseline and metabolic characteristics of the GDM-GH/PE group and the GDM group.

	GDM group n = 41	GDM-GH/PE group n = 41	P value
FABP4, ng/ml	12.79(±6.04)	17.53(±11.35)	0.020*
<9.74 ng/ml	14	6	
9.74–18.46 ng/ml	21	21	
>18.46 ng/ml	6	14	
Age, y	33.46(±4.03)	33.49(±4.24)	0.979
Gravidity	2(2–3)	2(1–3)	0.183
Parity	0(0–1)	0(0–1)	0.330
Pre-pregnancy BMI, kg/m^2^	24.73(±4.60)	25.59(±4.66)	0.402
Second trimester BMI, kg/m^2^	27.43(±4.79)	28.75(±4.94)	0.222
Gestational age, wks	27.78(±1.19)	27.93(±1.71)	0.633
OGTT-fasting glucose, mmol/l	5.25[4.90–5.43]	5.26[4.81–5.56]	0.860
OGTT-1 hour glucose, mmol/l	9.65(±1.60)	10.09(±1.59)	0.208
OGTT-2 hour glucose, mmol/l	8.29(±1.37)	8.60(±1.53)	0.338
TG, mmol/l	2.60[2.30–2.98]	3.13[2.43–3.85]	0.017*
TCHO, mmol/l	6.08(±1.07)	6.20(±1.17)	0.654
HDL, mmol/l	1.83(±0.38)	1.88(±0.39)	0.519
LDL, mmol/l	3.14(±0.86)	3.16(±0.84)	0.916
HbA1c, %	5.43(±0.31)	5.50(±0.31)	0.327
HOMA-IR	2.54(±1.54)	3.37(±1.71)	0.024*
Second trimester SBP, mmHg	118(±13)	131(±10)	<0.001*
Second trimester DBP, mmHg	68(±9)	78(±9)	<0.001*
Second trimester MAP, mmHg	85.1(±9.6)	95.5(±8.0)	<0.001*

Data was presented as mean (±SD) or median (range).

* P<0.05

### Plasma FABP4

**Fig 1:**
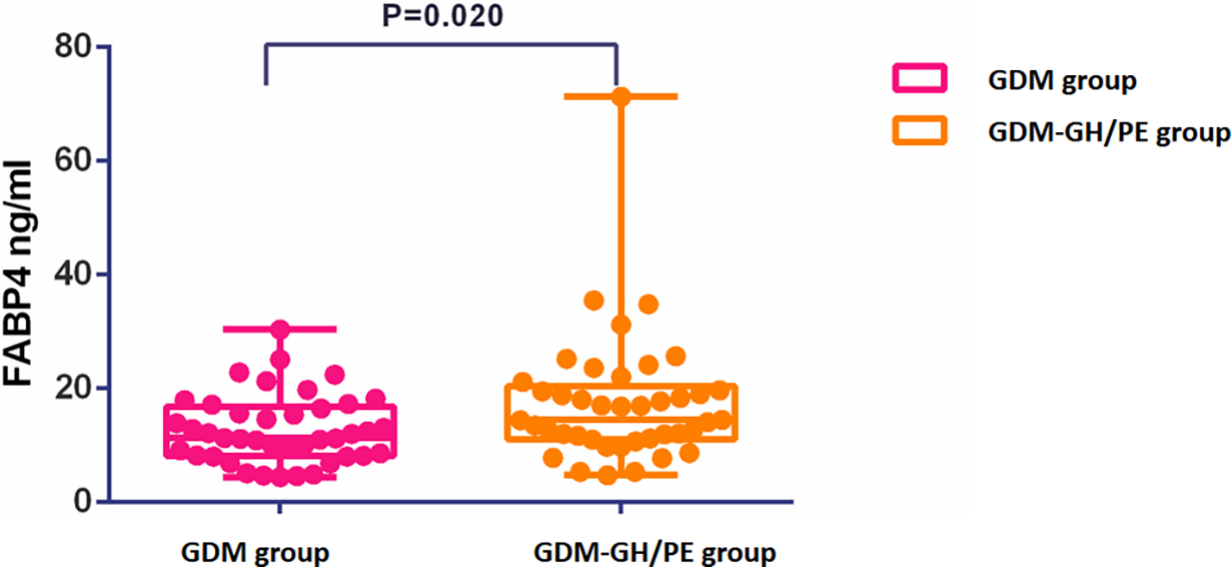
Comparison of fasting plasma FABP4 between GDM-HP group and GDM-PE group.

The level of plasma FABP4 in the GDM-GH/PE group was significantly higher than in the GDM group (17.53 ± 11.35 vs. 12.79 ± 6.04 ng / ml, P = 0.020) (Fig 1). The AUC for the second trimester plasma FABP4 alone predicted in GH/ PE in GDM patients was 0.647 (95% CI 0.529–0.766). Plasma FABP4 levels stratify the risk of developing GH/PE in GDM women, to some degree. When FABP4 levels were greater than 18.46 ng/ml (75^th^ percentile), 70% developed GH/PE, whereas when FABP4 was below 9.74 ng/ml (25^th^ percentile), only 30% developed GH/PE.

### Univariate correlations of plasma FABP4 with clinical and metabolic parameters

The univariate correlations of second trimester plasma FABP4 with BMI, glucose and lipid metabolism, and other clinical parameters are shown in Table 2. We found a modest correlation between maternal plasma FABP4 and BMI (both pre-pregnancy and second trimester) in both the GDM-GH/PE group and the GDM group. Univariate analysis of all cases also showed that FABP4 was weekly positively correlated with HOMA-IR, MAP, HbA1c, and gestational age. There was no correlation between FABP4 concentrations and the maternal age, gestational age, TG, TCHO, HDL, LDH, HbA1c, or MAP in neither the GDM-PE group nor the control group.

**Table 2:** Univariate correlations of plasma FABP4 with clinical and metabolic parameters.

	All patients	GDM group	GDM-GH/PE group
Age	-0.009/0.937	-0.072/0.654	0.045/0.779
Pre-pregnancy BMI	0.593/<0.001*	0.620/<0.001*	0.538/<0.001*
Second trimester BMI	0.584/<0.001*	0.587/<0.001*	0.546/<0.001*
Gestational weeks	0.236/0.033*	0.173/0.279	0.283/0.073
TG	0.184/0.097	0.093/0.562	0.130/0.418
TCHO	-0.012/0.915	-0.157/0.328	0.096/0.550
HDL	-0.066/0.553	-0.159/0.322	-0.047/0.768
LDL	-0.004/0.968	-0.151/0.346	0.125/0.435
HbA1c	0.239/0.030*	0.225/0.157	0.205/0.198
HOMA-IR	0.359/0.001*	0.338/0.031*	0.221/0.165
MAP	0.285/0.010*	0.240/0.130	0.096/0.551

Data are given for each analysis as assessed by Spearman’s correlation method (r/P values).

* P<0.05

### Logistic and multiple regression analysis

Multiple collinearity analysis was performed separately between each of the variables, including plasma FABP4, maternal age, pre-pregnancy BMI, second trimester BMI, gestational age, TG, TCHO, HDL, LDL, HbA1c, HOMA-IR, and MAP. All VIF values were less than 10, and tolerance for all were larger than 1. Thus, there was no significant collinearity among the variables, and the logistic regression analysis was performed subsequently.

Multivariate analysis showed that FABP4 was the independent risk factor for GH/PE in GDM patients (OR = 1.136, 95% CI 1.003–1.286, P = 0.045). The variables of the prediction model and their coefficients are shown in Table 3. The AUC was 0.890 (95% CI 0.818–0.962). When FABP4 was removed from the model containing established risk factors, the AUC was 0.886 (95%CI 0.809–0.964). The AUC of each model is summarized in Table 4.

**Table 3:** Multivariable logistic regression model examining the association among clinical factors and GH/PE in GDM women.

	coefficient	P	OR	95%CI
FABP4	0.127	0.045	1.136	1.003–1.286
MAP	0.190	<0.001	1.210	1.096–1.335
Second trimester BMI	-0.202	0.043	0.817	0.672–0.993
TG	1.950	0.003	7.029	1.966–25.133
TCHO	-1.932	0.082	0.145	0.016–1.277
HDL	3.301	0.029	27.141	1.400–526.132
LDL	2.535	0.034	12.614	1.209–131.640
Constant	-21.383	<0.001		

**Table 4:** Plasma FABP4 concentrations in the second trimester with AUC of each model.

	FABP4 alone	Established risk factors without FABP4	Established risk factors with FABP4
AUC(95%CI)	0.647(0.529–0.766)	0.886(0.809–0.964)	0.890(0.818–0.962)

### Subgroup analysis

**Fig 2:**
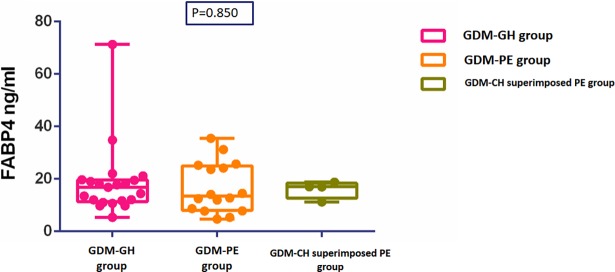
Comparison of fasting plasma FABP4 between GDM-GH group, GDM-PE and GDM-CH superimposed PE group.

The GH/PE group was then further divided into the GDM-GH group (n = 21), the GDM-PE group (n = 16) and GDM-CH superimposed PE group (n = 4) according to later developed gestational hypertension or preeclampsia. There was no significant difference in plasma FABP4 concentrations between the three groups (GDM-GH group 18.53±13.59 ng/ml, the GDM-PE group 16.61±9.60 ng/ml, GDM-CH superimposed PE group 16.01±3.3ng/ml, P = 0.850) (Fig 2).

## Discussion

Pregnant patients may develop the metabolic syndrome preceding or along the duration of pregnancy. Hypertensive disorders (including GH and PE) and gestational diabetes mellitus (GDM) are central attributes of the metabolic syndrome that may occur in pregnancy [18]. They have some similar risk factors, such as obesity and insulin resistance, and have long-term influences on women’s health. GDM itself is one of the risk factors for pregnancy hypertension and preeclampsia [19]. According to the literature reported over time and from different regions, the incidence of GH/PE in GDM women is 12% -17% [20, 21], and the incidence of PE in GDM women is 4%-8% [20, 22, 23]. The recent random sampling study of 15 hospitals in Beijing reported the incidence of GH/PE and PE at 6.2% and 3.7% in women with GDM, whereas only 3.8% and 2.4% in the unaffected population [24]. Several studies have shown that serum FABP4 levels of GDM women were higher than the levels found in women with normal pregnancies [25–28]. Considering the potential relationship between FABP4 and preeclampsia, and its current evidence, we believe that FABP4 may be associated with different phenotypes of metabolic syndrome in pregnancy. To our knowledge, there have been no reports about whether the level of FABP4 in GDM women would affect their risk to develop GH/PE, subsequently.

The incidence of GH/PE and PE was 6.44% and 3.30%, respectively, in our study, which is in accordance with the recent data from Beijing, but lower than previously reported in both China and other countries. This may be attributed to different GDM diagnostic criteria, different ethnic characteristics, the standardized prenatal care, and more attention and management to hyperglycemia in pregnancy, especially the GDM one-day clinic in our hospital. Our research was conducted in a case-control study due to low incidence.

Our study showed that the plasma FABP4 concentration was significantly higher in the GDM-GH/PE group than in the control group, even after adjustment for the maternal age, BMI, blood lipid, HbA1c, HOMA-IR, and blood pressure, which indicated that increased second trimester plasma FABP4 independently predicted GH/PE in GDM patients. But its individual predictive value is limited with an AUC of 0.647 (95% CI 0.529–0.766). On the other hand, FABP4 level, when added to established risk factors, did not significantly increase the AUROC (the AUC for established risk factors without FABP4 and with FABP4 were 0.886 and 0.890, respectively). Thus, whether FABP4 was a superior predictor factor of GH/PE in GDM patients remains to be further explored.

A previous study reported that maternal FABP4 concentrations were elevated in preeclampsia [11], even before its clinical onset in both the normal population [11] and women with T1DM [13]. A study focused on overweight patients showed that serum FABP4 concentrations in the second trimester were associated with subsequent development of GH/PE (refer to pregnancy induced hypertension (PIH) in the study) [12]. However, the subgroup analysis of this study showed that there was no difference of maternal FABP4 between the preeclampsia group and control group, which may due to limited sample size. In the subgroup analysis of our study, there was no difference of maternal FABP4 levels between the GH subgroup and PE subgroup. This may reveal that potential metabolic causes may contribute to the mechanism of gestational hypertension and preeclampsia. FABP4 is a potential predictive factor of the subsequent GH/PE in GDM patients, but it may not be specific for predicting preeclampsia.

Our study showed that plasma FABP4 levels were correlated with BMI, HOMA-IR, HbA1c, and MAP to varying degrees. This reflects that the level of FABP4 itself is associated with glycolipid metabolism, insulin resistance and other metabolic factors. It was already confirmed that in the non-gestational population, FABP4 is an independent risk factor, not only for not metabolic syndrome but also its related indexes, including waist circumference, blood pressure, dyslipidemia, and insulin resistance [6, 29]. Interestingly, as a member of adipokines, the level of FABP4 showed no significant correlation with the lipid level, including TG, TCHO, HDL, and LDH. A previous study showed a positive correlation between TG and FABP4 levels in GDM women. This may be contributed to different populations and the severity of disease status [26].

Our study also showed that SBP, DBP and MAP in GDM women that later develop GH/PE were significantly higher than those that did not. This suggests that when we established a predictive model of gestational hypertension or preeclampsia, the baseline blood pressure of the patient should be included as a potential candidate. Considering the strong linear relationship among SBP and DBP, MAP maybe an ideal index to represent their baseline blood pressure.

The HOMA-IR of the GDM-GH/PE group was significantly higher than that of GDM group, as well as the level of TG. This suggests that insulin resistance and dyslipidemia may be associated with the development of GH/PE in GDM. What should be noted is that HOMA-IR appeared not to be an independent predictor for GH/PE in our study, which may be related to the inclusion of index of glucolipid metabolism, and FABP4.

Our research has limitations. First, although our study found elevated plasma FABP4 in GDM women that later developed GH/PE, it could not prove whether FABP4 played a role in the pathogenesis because of its observational characteristics. Then, as a case-control study, selected bias may exist, thus the evidence level was lower than that of the cohort study. In addition, it is difficult to determine the predictive threshold due to the limited sample size, and it made it difficult to evaluate its positive and negative predictive values. The small number of women also limited our statistical significance. Further studies are needed with larger sample sizes, within the whole cohort of the population, and may include examining FABP4 levels in different stages of pregnancy, especially postpartum, and then conducting a long-term follow-up to explore whether postpartum FABP4 levels play a role in predicting future metabolic syndrome and cardiovascular disease.

## Conclusions

In conclusion, our study suggested that increased second trimester plasma FABP4 independently predicted GH/PE in GDM patients.

## Supporting information

S1 File Original data-variability of the FABP4. This file includes the original data regarding the variability of the FABP4 assay used.
(XLSX)
